# Genome-wide identification and classification of *MYB* superfamily genes in peach

**DOI:** 10.1371/journal.pone.0199192

**Published:** 2018-06-21

**Authors:** Chunhua Zhang, Ruijuan Ma, Jianlan Xu, Juan Yan, Lei Guo, Juan Song, Ruchao Feng, Mingliang Yu

**Affiliations:** Institute of Pomology, Jiangsu Academy of Agricultural Sciences/Jiangsu Key Laboratory for Horticultural Crop Genetic Improvement, Nanjing, Jiangsu, China; Wuhan Botanical Garden, CHINA

## Abstract

The MYB transcription factor superfamily is one of the largest superfamilies modulating various biological processes in plants. Over the past few decades, many *MYB* superfamily genes have been identified and characterized in some plant species. However, genes belonging to the MYB superfamily in peach (*Prunus persica*) have not been comprehensively identified and characterized although the genome sequences of peach were released several years ago. In total, this study yielded a set of 256 *MYB* superfamily genes that was divided into five subfamilies: the R2R3-MYB (2R-MYB), R1R2R3-MYB (3R-MYB), MYB-related (1R-MYB), 4R-MYB, and Atypical-MYB subfamilies. These subfamilies contained 128, 4, 109, 1, and 14 members, respectively. The 128 R2R3-MYB subfamily genes in peach were further clustered into 35 groups, and the 109 MYB-related subfamily genes were further clustered into 6 groups: the CCA1-like, CPC-like, TBP-like, I-box-binding-like, R-R-type, and Peach-specific groups. The motif compositions and exon/intron structures within each group within the R2R3-MYB or MYB-related subfamily in peach were highly conserved. The logo sequences of the R2 and R3 repeats of R2R3-MYB subfamily members were highly conserved with those in these repeats of several other plant species. Except for 48 novel peach-specific *MYB* genes, the remaining 208 out of 256 *MYB* genes in peach were conserved with the corresponding 198 *MYB* genes in *A*. *thaliana*. Additionally, the 256 *MYB* genes unevenly distributed on chromosomes 1 to 8 of the peach genome. Eighty-one orthologous pairs of peach/*A*. *thaliana MYB* genes were identified among 256 *MYB* genes in peach and 198 *MYB* genes in *A*. *thaliana* in this study. In addition, 146 pairs of paralogous *MYB* genes were identified on the eight chromosomes of peach. The expression levels of some of the 51 *MYB* genes selected for qRT-PCR analysis decreased or increased with red-fleshed fruit development, while the expression patterns of some genes followed no clear rules over the five developmental stages of fruits. This study laid the foundation for further functional analysis of *MYB* superfamily genes in peach and enriched the knowledge of *MYB* superfamily genes in plant species.

## Introduction

Transcription factors (TFs) control gene expression, and they play key roles in the regulation of cell activities [1,2]. Therefore, changes in the expression of TF genes can eventually lead to dramatic alterations in a plant [3]. TFs are usually dispersed throughout the genome of a plant species. Thus, the recent shift from gene-centric to genome-centric approaches in biology is appreciated for exploring TFs [4]. The majority of TFs can be classified into different gene families according to the conserved domains that are shared by all the members within a given family and specifically bind to DNA sequences in the regulatory regions of downstream target genes [4]. Among the various TF families in the genome, the MYB family is one of the largest and functionally diverse families [5]. Thus, it is usually called the MYB superfamily. MYB superfamily proteins are characterized by having a highly conserved MYB domain at the N-terminus that is usually composed of 1–4 adjacent imperfect tandem repeats (R1-R4) [6]. Each imperfect tandem repeat encodes 50–53 amino acid residues and contains three α-helices, the second and third of which form a helix-turn-helix (HTH) motif [7]. Based on the number of MYB imperfect tandem repeat (R), MYB superfamily members are classified into four subfamilies, namely, MYB-related proteins (or 1R-MYB proteins, having one R), R2R3-MYB proteins (having two Rs), R1R2R3-MYB proteins (having three Rs), and 4R-MYB proteins (having four Rs) [6,8]. The R1R2R3-MYB subfamily proteins are predominant in animals, but the R2R3-MYB subfamily proteins are the most prevalent in plants [6]. Based on their conserved domains and phylogenetic relationships, R2R3-MYB subfamily proteins have been classified into 30–38 groups in plants [6,8]. As an increasing number of genome sequences have been released, a variety of MYB superfamily proteins have been identified and analyzed in a wide range of plant species such as sesame [6], *Arabidopsis thaliana* (*A*. *thaliana*) [7], *Brassica napus* (*B*. *napus*) [8], kiwi [9], *Brachypodium distachyon* (*B*. *distachyon*) [10], foxtail millet [11], and pear [12]. Furthermore, multiple MYB proteins in plants have been functionally studied in detail. The results from these studies have proved that they are involved in regulating a wide range of intricate networks, growth, development, and stress resistancethroughout the plant life cycle, including the anthocyanin biosynthetic pathway [13,14], the coloration of fruit skin [14], flavonoid/phenylpropanoid metabolism [15], secondary wall biosynthesis [16], sugar signaling [17], and responses to drought and waterlogging stress [6].

Peach (*Prunus persica*) is an important horticultural fruit and a model plant of Rosaceae family with characteristics such as a short life cycle, the ability to self-pollinate and a small genome size of 265 Mb [18]. Peach is native to China. Although there is a wide range of cultivated peach cultivars in China, there is only four kinds of flesh color in general: white, green, yellow and red. In recent decades, red-fleshed peaches attracted a lot of attention from breeders and consumers worldwide, because of the exceptional colors and functional components enrichment. The molecular mechanisms underlying red-fleshed trait is still not clear in peach. It is highly important to explore genes involved in the regulation of red color formation in peach for accelerating the breeding progress of red-fleshed peach cultivars. In some fruit tree species, anthocyanin have been reported to involve in the formation of red flesh color, and *MYB* genes have been suggested to be involved in the biosynthesis of anthocyanin [19,20]. Recently, whole genome sequences of peach were added to the Genome Database for Rosaceae (GDR), providing an important foundation for the genome-wide identification of genes of a given peach family [21,22]. Genes of the peach MYB superfamily have not been comprehensively identified and characterized. Thus, the identification and characterization of all *MYB* superfamily genes and their subfamily and group classification, motif and exon/intron composition, syntenic relationships, and expression levels were analyzed in this study. The findings of this study will be helpful for further functional studies of interesting *MYB* superfamily genes involved in fruit development in peach.

## Materials and methods

### Plant materials

The fruits from one-year-old fruiting shoots were selected as experimental materials from the outer southern canopies of six-year-old ‘Zhaoxia’ (ZX, white flesh) and ‘Yejihong’ (YJH, red flesh) peach trees grown under the same field conditions and standard management at the National Peach Germplasm Repository in Nanjing, China. Nine trees of each cultivar were divided into three plots from which three trees per plot were selected for sample collection. The fruits from the two aforementioned cultivars were picked 51, 64, 75, 84, and 93 days after full bloom (DAFB) and were immediately frozen in liquid nitrogen, followed by storage at -80 °C. For quantitative real-time PCR (qRT-PCR), three technical repeats were performed for each sample at each developmental stage.

### Identification of peach *MYB* superfamily genes

The Hidden Markov Model (HMM) profile for the MYB binding domain (PF00249) obtained from the Pfam database (http://pfam.xfam.org/) [23] was used as a query to search peach genome versions (v) 1.0 and 2.0 using HMMER (http://hmmer.org/) for selecting ID numbers of all sequences containing MYB or MYB-like domains in peach, respectively. The corresponding protein sequence for each ID number obtained from the HMMER results was downloaded from Phytozome [20] and the GDR websites. The protein sequences were then submitted to the Conserved Domain search (CD search) [24] and InterProScan [25] to further ensure the presence of the conserved MYB domain. The sequences with MYB domains were considered the final MYB superfamily members. Subsequently, the transcript sequences, coding sequences (CDSs), genome sequences, chromosome locations, and predicted functions corresponding to these final MYB superfamily proteins were downloaded from the Phytozome and GDR databases. Additionally, 198 MYB superfamily protein sequences and chromosome locations in *A*. *thaliana* were obtained from The Arabidopsis Information Resource (TAIR; http://www.arabidopsis.org/) based on previous work [26,27].

### Construction of phylogenetic trees and conserved motif analysis of MYB proteins

To classify the peach MYB proteins into groups and identify the evolutionary relationships between the MYB proteins of peach and *A*. *thaliana*, phylogenetic trees were constructed using the sequences of the peach MYB proteins and *A*. *thaliana* MYB proteins. The amino acid sequences of the 126 R2R3-MYB proteins and 5 R1R2R3-MYB proteins in *A*. *thaliana* were downloaded from the TAIR database [26]. The first phylogenetic tree was constructed using the 126 R2R3-MYB-subfamily proteins and 5 R1R2R3-MYB-subfamily proteins of *A*. *thaliana* and all 256 MYB superfamily proteins of peach. The multiple alignment of the R2R3-MYB and R1R2R3-MYB amino acid sequences of peach and *A*. *thaliana* was performed using the ClustalW program [28]. The neighbor-joining phylogenetic tree was constructed with a bootstrap analysis of 1,000 replicates using the MEGA 4.1 software. The MYB subfamily classification of *A*. *thaliana* genes [7,29] and the first phylogenetic tree of peach and *A*. *thaliana* in this study were used as references to classify the R2R3-MYB and R1R2R3-MYB subfamilies in peach into groups.

The 124 proteins of the MYB superfamily that did not belong to either of the R2R3-MYB or R1R2R3-MYB subfamilies in the first phylogenetic tree, in addition to 60 1R-MYB proteins, 1 4R-MYB protein, and 6-Atypical-MYB proteins in *A*. *thaliana* [27], were used to construct the second phylogenetic tree. The parameters were set as described for the first phylogenetic tree. Based on previous work [27,30], the 124 proteins of the MYB superfamily in peach were subdivided into several other groups.

Conserved motifs shared by MYB proteins were analyzed using Multiple Em for Motif Elicitation (MEME v4.12.0, http://meme.nbcr.net/meme/cgi-bin/meme.cgi) online tool [31] through uploading the amino acid sequences of the MYB superfamily members in peach. The following parameter settings were used: 0 or 1 for the occurrence of a single motif per sequence, motif width ranges of 2 to 250 amino acids, and 15 as the maximum number of motifs that could be found. All other parameters were default settings. The orders of the ID numbers of MYB proteins in the first and second motif figures are the same as those in the first and second phylogenetic trees, respectively. The physiological and biochemical characteristics of MYB proteins in peach were analyzed using the ExPASy online tool (http://www.expasy.ch/tools/protparam.html).

### Analysis of the characteristics of *MYB* genes

The length of genomic sequences, CDSs, transcript sequences, and protein sequences and the genome locations and ID numbers (peach genome v1.0 and v2.0) of peach MYB members were downloaded from the Phytozome database. Combined genome location with the length of each chromosome obtained from GDR website, peach *MYB* genes were mapped to corresponding location on the eight chromosomes using the circlize package of R (v0.4.3). The genome location of each MYB member and the length of each chromosome of *A*. *thaliana* was obtained from the TAIR database. Based on above information, the orthologous *MYB* genes between peach and *A*. *thaliana*, and the paralogous genes in peach or *A*. *thaliana* were analyzed using OrthoMCL software [32], followed by the drawing of a synteny figure using the Circos tool [33]. The exon/intron structures of peach *MYB* genes were plotted using the Gene Structure Display Server (GSDS) (http://gsds.cbi.pku.edu.cn/) [34]. The orders of the ID numbers of *MYB* genes in the first and second exon/intron figures are the same as those in the first and second phylogenetic trees, respectively.

### Analysis of peach *MYB* superfamily gene expression

RNA isolation from frozen sampled fruits of the two cultivars was carried out using the Plant RNA Kit (TaKaRa Biotechnology Co. Ltd., Dalian, China). The high-quality total RNA was then reverse transcribed to cDNA using the PrimeScript^™^ RT Reagent Kit with gDNA Eraser (TaKaRa Biotechnology Co. Ltd., Dalian, China). All cDNA samples were adjusted to 100 ng μl^−1^ with sterilized distilled water and were used as templates for qRT-PCR.

According to previous work [6], R2R3-MYB subfamily proteins are the most prevalent in plants. Therefore, we selected 49 genes from groups C3 to C24 and C31, and C32 of R2R3-MYB subfamily to analyze the expression characteristics and predict the function. The R1R2R3-MYB subfamily proteins have been reported to be predominant in animals [6], and to analyze the relative expression levels, 2 genes from the R1R2R3-MYB subfamily were also selected. For qRT-PCR analysis, gene-specific primers for each of the 51 *MYB* genes were designed based on the CDSs of the peach genome v2.0 using Primer Premier 5.0 software (Premier Biosoft) (S1 Table). Each pair of primers was designed to avoid being located within the conserved region to guarantee specificity of primer pair. *RNA polymerase II* (*RP II*, accession number TC1717) of peach were used as housekeeping gene for normalization in this study [35]. qRT-PCRs were performed on an Applied Biosystems 7500 Real-Time PCR system using SYBR^®^ Premix Ex Taq^™^ reagent (Tli RNaseH Plus) (TaKaRa Biotechnology Co. Ltd., Dalian, China) according to the manufacturer’s instructions. Each reaction mixture (20 μl) contained 1.0 μl of diluted cDNA, 0.4 μl of each primer, 0.4 μl of ROX, 10.0 μl of master mix, and 7.8 μl of RNase-free water. Thermal cycling conditions were set as per the manufacturer’s instructions for SYBR^®^ Premix Ex Taq^™^. Each assay was repeated three times biologically using replicate fruit samples. The relative changes in gene expression were calculated using the 2^-ΔΔ*C*^_T_ method [36].

## Results and discussion

### Identification of peach *MYB* superfamily genes

Based on the HMM results, 256 and 234 *MYB* genes were predicted for the MYB superfamily in peach genome v1.0 and v2.0, respectively (S2 Table). The CDSs of 234 *MYB* genes in peach genome v2.0 were identical to those of the corresponding 234 genes in peach genome v1.0; The corresponding ID numbers and sequences for the remaining 22 genes with ID numbers in peach genome v1.0 were not found within peach genome v2.0 (S2 Table). In summary, 256 candidate genes for the MYB superfamily were identified in peach. All 256 MYB proteins had typical MYB or MYB-like domains. Combined with the number of *MYB* genes identified in other plant species in previous studies, this finding in peach is consistent with the MYB superfamily being one of the largest TF superfamilies in plants [5,24]. The MYB members have been studied in diverse species and particularly in plants. Many *MYB* genes have been identified and characterized in some plant species: 287 *MYB* genes were found in sesame [6], 122 in *B*. *distachyon* [10], 127 in *Solanum lycopersicum* [37], and 177 in sweet orange [30]. The differences in the total numbers of MYB superfamily members are probably associated with the differences in genome sizes and evolution of these plants.

The ID numbers of the peach *MYB* genes from both peach genomes v1.0 and v2.0 are listed in S2 Table. The lengths of the protein sequences of the 256 peach *MYB* genes ranged from 37 to 2405 amino acids. The lengths of the CDSs of the 256 peach *MYB* genes ranged from 201 to 6138 bp. The lengths of the genomic sequences of the 256 peach *MYB* genes ranged from 279 to 13961 bp. The lengths of the transcript sequences of the 256 peach *MYB* genes ranged from 279 to 7315 nt. Most of these genes were predicted to be MYB TFs.

The protein sequence of each peach MYB member was submitted to the BLAST tool in the TAIR database to search for the *MYB* genes in *A*. *thaliana* that were most homologous (S2 Table). The result showed that some peach *MYB* genes had a homologous gene in *A*. *thaliana*; for example, Prupe.2G158300.1, Prupe.1G452000.1, and Prupe.5G093400.1 were all homologous to AT4G32730.2. Other peach genes that shared an *A*. *thaliana* homolog included the pair of Prupe.2G252700.1 and Prupe.8G134900.1 and the pair of Prupe.1G517400.1 and Prupe.6G188300.1.

### Phylogenetic trees and group classification of MYB proteins

**Fig 1:**
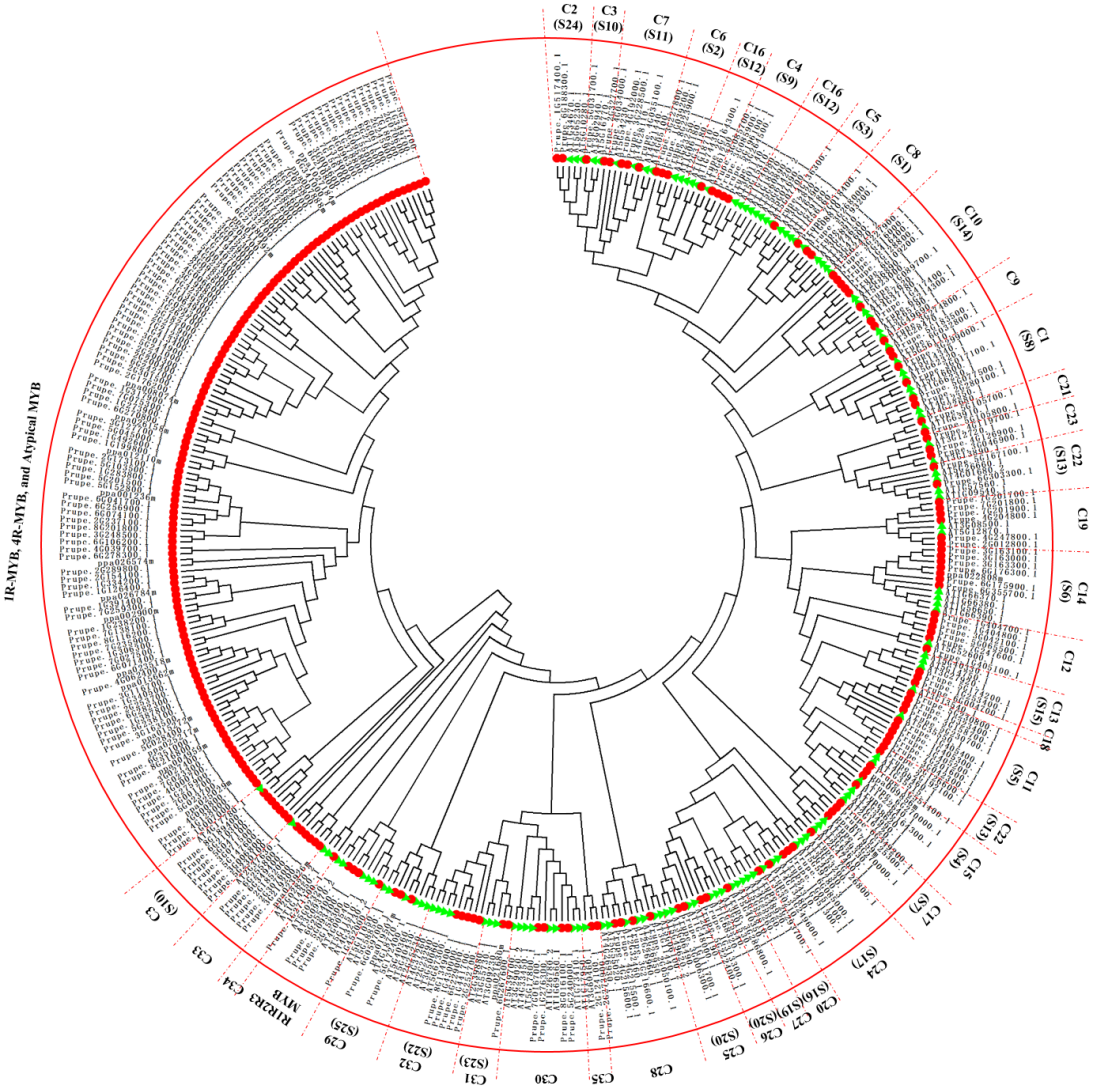
Phylogenetic tree of R2R3-MYB and R1R2R3-MYB (3R-MYB) subfamilies of *A*. *thaliana* and peach MYB proteins. The phylogenetic tree was constructed for R2R3-MYB and R1R2R3-MYB subfamilies using the sequences of 131 R2R3-MYB and R1R2R3-MYB proteins in *A*. *thaliana* and 256 MYB proteins of peach. The small red circles represent the 256 peach MYB proteins; the small green triangles represent the 126 R2R3-MYB subfamily and 5 R1R2R3-MYB subfamily proteins from *A*. *thaliana*. The English letters with Arabic numbers outside the large red circle indicate the names of each group of the peach R2R3-MYB and R1R2R3-MYB subfamilies. The red dotted line represents the initial or final boundary of each group of the peach R2R3-MYB and R1R2R3-MYB subfamilies.

**Fig 2:**
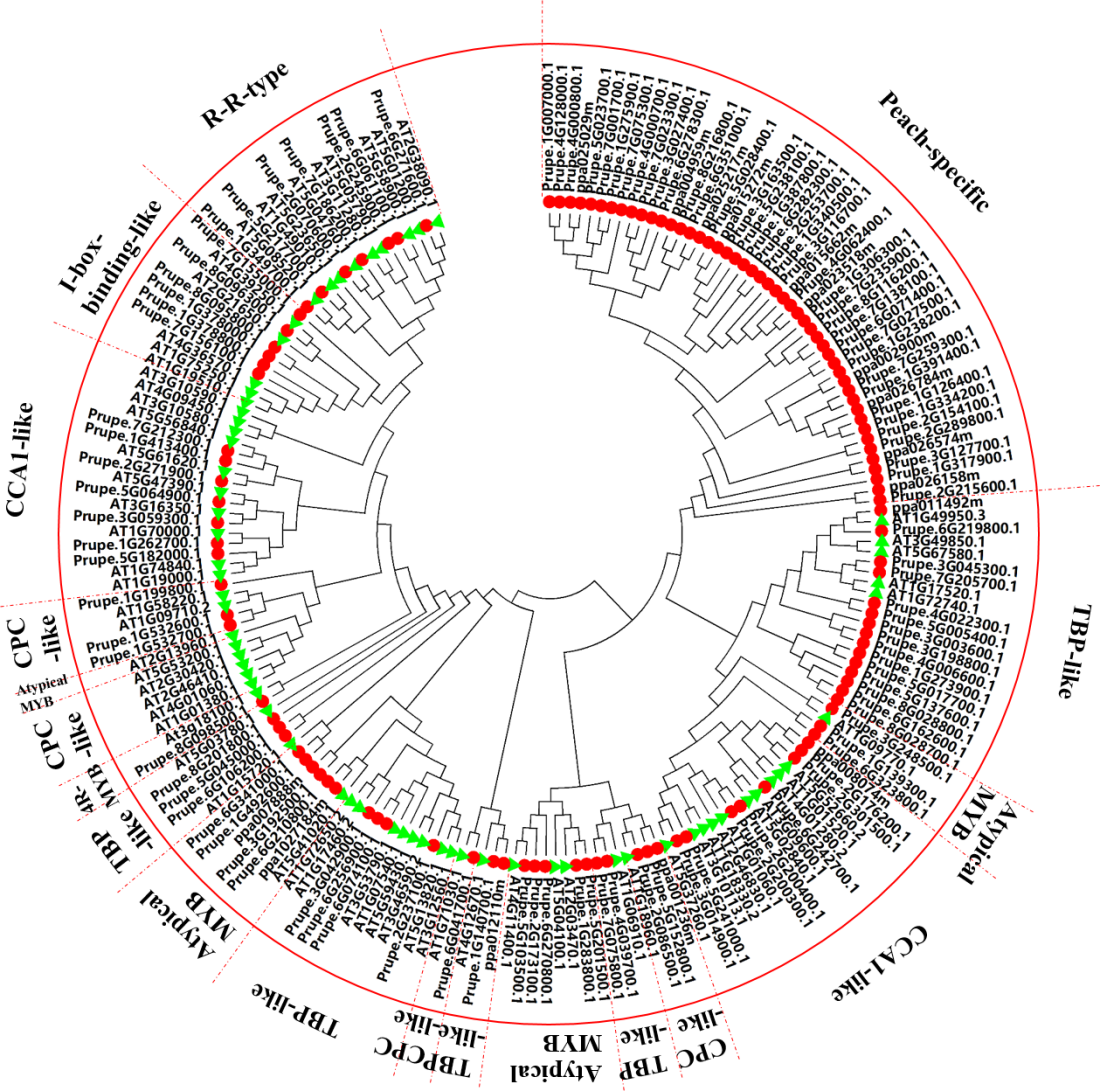
Phylogenetic tree and classification of MYB-related (1R-MYB), 4R-MYB, and Atypical-MYB subfamily proteins in *A*. *thaliana* and peach. The phylogenetic tree was constructed using the sequences of 60 MYB-related, 1 4R-MYB, and 6 Atypical-MYB proteins in *A*. *thaliana* and the 124 remaining peach MYB proteins that did not belong to any subfamily of the first phylogenetic tree. The small red circles represent the 124 peach MYB proteins; the small green triangles represent the 67 MYB proteins of *A*. *thaliana*. The English letters with Arabic numbers outside the large red circle indicate the names of each group of the peach. The red dotted line represents the initial or final boundary of each group of the peach.

In summary, this study yielded a set of 256 MYB proteins containing 128 R2R3-MYB proteins (2R-MYB), 4 R1R2R3-MYB proteins (3R-MYB), 109 MYB-related proteins (1R-MYB), one 4R-MYB protein, and 14 Atypical-MYB proteins (Figs 1 and 2). These results revealed that 4R-MYB type consisted of the lowest number of MYB proteins, with 0.4% (1 protein) of the total MYB proteins. Similarly, only 4 3R-MYB type proteins (1.56%) and 14 Atypical-MYB type proteins (5.47%) were identified in this study. In contrast, the 1R-MYB proteins accounted for 42.58% (109 proteins) of the total peach MYBs and thus constituted the second largest group of MYB proteins. The 128 R2R3-MYB proteins accounted for half (50%) of the total MYB proteins were thus the largest subfamily of MYB superfamily in peach. This result is consistent with previous reports; in plants, the R2R3-MYB subfamily is the most abundant transcription factor subfamily [27], with 126 R2R3-MYB members in *A*. *thaliana* [22,23], 121 in tomato [38], 85 in sweet orange [30], 93 in kiwi [9], 222 in apple [39], and 192 in *Populus* [40].

Because the 256 peach MYB proteins and 198 *A*. *thaliana* MYB proteins were too numerous for each gene ID to be clearly seen in the circle of a phylogenetic tree, two phylogenetic trees were constructed in this study. The first phylogenetic tree (Fig 1) was composed of the 131 MYB proteins belonging to subfamilies R2R3-MYB (126 members) and R1R2R3-MYB (5 members) in *A*. *thaliana* (S3 Table) and 256 peach MYB proteins. As a result, 128 of 256 peach MYB proteins were clustered into the R2R3-MYB subfamily with 126 *A*. *thaliana* MYB proteins. These 128 peach R2R3-MYB members were subdivided into 35 groups based on the topology of the tree and the classification of the MYB superfamily in *A*. *thaliana* and pear [7,29]. The 126 and 128 members of R2R3-MYB subfamilies from *A*. *thaliana* (marked by green triangles) and peach (marked by red circles), respectively, were well separated into 35 different groups. The 35 groups were designated C1 to C35 (Fig 1). These groups were different from the groups reported by previous phylogenetic analyses of other plant species although each group had the same name, such as sesame [6], *Solanum lycopersicum* [37], sweet orange [30]. In those references, each group was named according to the occurrence order in the topology in the circular phylogenetic tree. The number of peach R2R3-MYB members in each group ranged from 1 to 9 in this study. In terms of the total numbers of groups subdivided from the R2R3-MYB subfamily, the R2R3-MYB subfamily can usually be divided into 18 to 48 groups based on previous work [41]. In kiwi, sweet orange, and tomato, the R2R3-MYB subfamily was subdivided into 36, 30, and 29 groups, respectively [9,30,38].

Based on the classification of the R1R2R3-MYB (3R-MYB) subfamily in *A*. *thaliana*, 4 of 256 peach MYB proteins, Prupe.1G452000.1, Prupe.2G158300.1, Prupe.5G093400.1, Prupe.6G255400.1, were clustered into the R1R2R3-MYB subfamily with five *A*. *thaliana* members (Fig 1). This number was similar to previous findings in sesame [6], tomato [37], and *B*. *distachyon* [10], which had 5, 4, and 3 members in the R1R2R3-MYB subfamily, respectively. However, 10 members were identified in the R1R2R3-MYB subfamily for Chinese white pear [29]. The findings in this study verified the previous description for the MYB superfamily, which stated that R1R2R3-MYB proteins are dominantly owned by animals but the R2R3-MYB proteins are enriched in plants [7,42].

In contrast to the R2R3-MYB subfamily, the MYB-related (1R-MYB) subfamily has attracted little attention, and only the MYB-related (1R-MYB) subfamily of a few plant species has been studied to date. In this study, in order to give a comprehensive result for all of the *MYB* genes in peach, a second phylogenetic tree (Fig 2) was constructed using 1 4R-MYB protein, 6 Atypical-MYB proteins, and 60 1R-MYB proteins in *A*. *thaliana* and the remaining 124 peach MYB proteins located at the end of the first phylogenetic tree. *A*. *thaliana* was used as the reference for classification [27], and the peach 1R-MYB subfamily members clustered with the 1R-MYB proteins in *A*. *thaliana* to be classified into six groups: CCA1-like, CPC-like, TBP-like, I-box-binding-like, R-R-type, and Peach-specific groups (Fig 2). The TBP-like group, with 26 members, is the second largest of the six groups, and its members dispersed throughout the five clades around the circle of the phylogenetic tree. Almost all of the members of this group contain the same conserved motif. The CCA1-like and CPC-like groups, with 15 and 7 members, distributed throughout two and three parts around the circle of the phylogenetic tree, respectively. This arrangement is consistent with that in *A*. *thaliana* and rice, where the CPC-like group also separately clustered into three clades, but it is different from that in sweet orange, whose CPC-like group clustered into two clades [30]. Consistent with Chen et al. [27] but different from Reichinann and Ratcliffe’s [43] classification, the I-box-binding-like group was divided into two groups in peach, namely, the I-box-binding-like group and the R-R-type group. On the one hand, the motif composition was the same for the members of each group (I-box-binding-like or R-R-type) but different between the I-box-binding-like and R-R-type groups. On the other hand, the lengths of the protein sequences within the I-box-binding-like group were all similar and relatively short, and the lengths of the protein sequences within the R-R-type group were similar but longer than those of all the members of the I-box-binding-like group. These features indirectly confirmed the correct classification for the 1R-MYB subfamily in peach.

Within the second phylogenetic tree (Fig 2), only one 4R-MYB protein (Prupe.8G098500.1) was identified in this study, the same number as in tomato [37], sweet orange [30], pear [29], and *A*. *thaliana* [7]. This finding is consistent with a previous report that found a single 4R-MYB protein encoded in several plant genomes [7]. In contrast, no 4R-MYB protein was identified in *B*. *distachyon* [10] and sesame [6]. Additionally, 48 sequences that did not belong to any above subfamilies remained at the beginning of the second phylogenetic tree and assigned to the Peach-specific group in peach in this study (Fig 2). No MYB protein from *A*. *thaliana* was clustered with those 48 Peach-specific MYB proteins, which were located at the beginning of clade of the second phylogenetic tree. This result means that not all the identified peach MYB TFs had a corresponding representative gene in *A*. *thaliana* and suggested that these peach proteins might have fruit-related functions that were either not needed in *A*. *thaliana* or were acquired after divergence from the last common ancestor. The same case was also observed in sweet orange [30] and other plant species [38]. The author of a study on sweet orange clustered those genes into the 1R-MYB subfamily [30]. In this study, we also clustered them into the 1R-MYB subfamily, based on the repeat number. The detailed specific functions of these members need to be studied further. The Atypical-MYB group has 14 members, all with a conserved motif, and they were distributed in three branches around the circle of phylogenetic tree.

### Motif composition of MYB proteins

To investigate the features of the homologous domains and the frequencies at which the most prevalent amino acids at each position were found within each repeat of the peach MYB domains, we used the online MEME tool to search for conserved motifs shared by these proteins by uploading the amino acid sequences.

**Fig 3:**
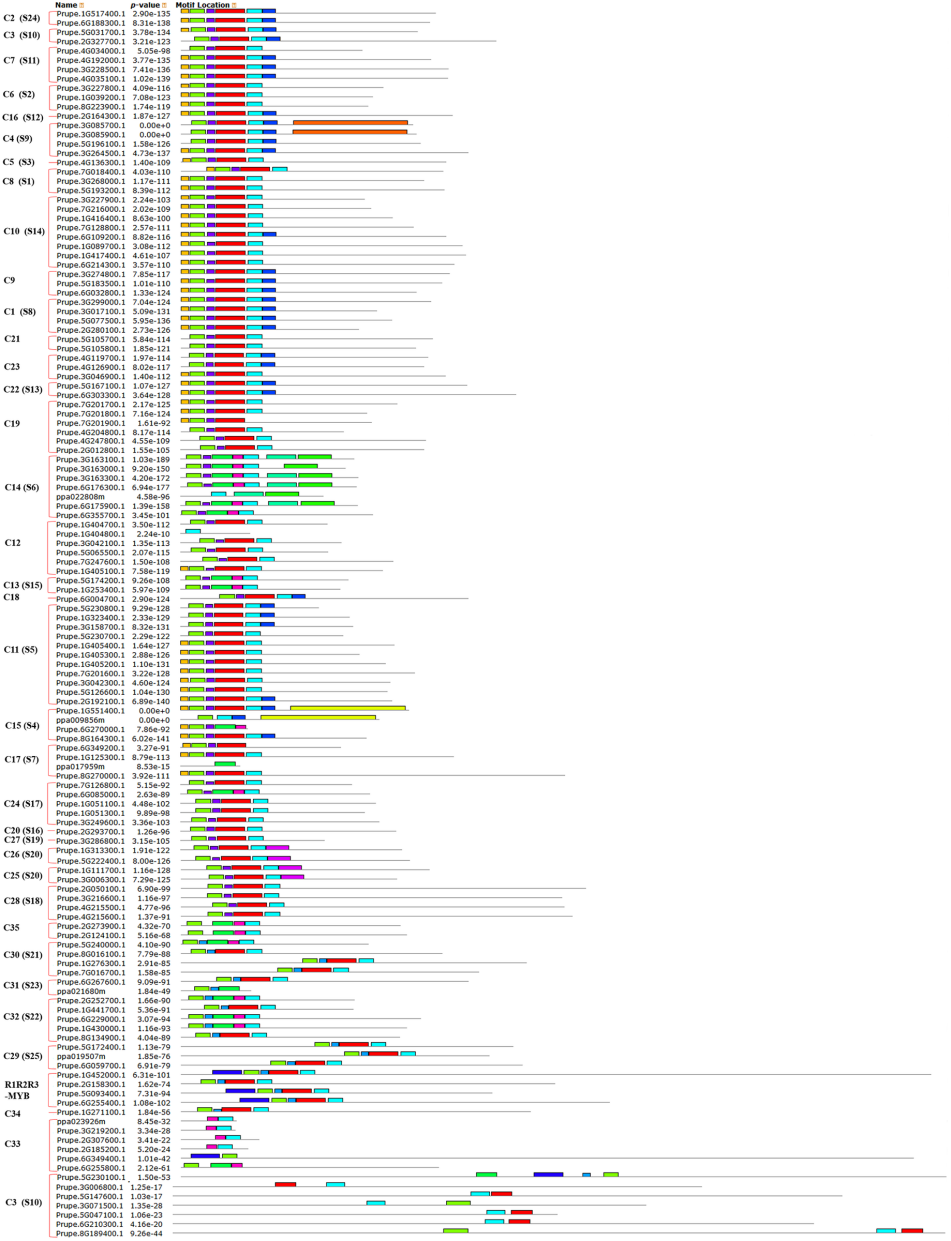
Motif distribution of peach R2R3-MYB subfamily and R1R2R3-MYB (3R-MYB) subfamily proteins. Motifs of the R2R3-MYB and R1R2R3-MYB subfamily proteins were analyzed using the MEME web server. Fifteen kinds of colored blocks represent 15 kinds of motifs. The length of the gray line indicates the length of a sequence relative to that of all the other sequences. The position of each block indicates the location of a motif with a matching sequence. The red brace on the right marks the members of each group of the R2R3-MYB and R1R2R3-MYB subfamilies in peach.

**Fig 4:**
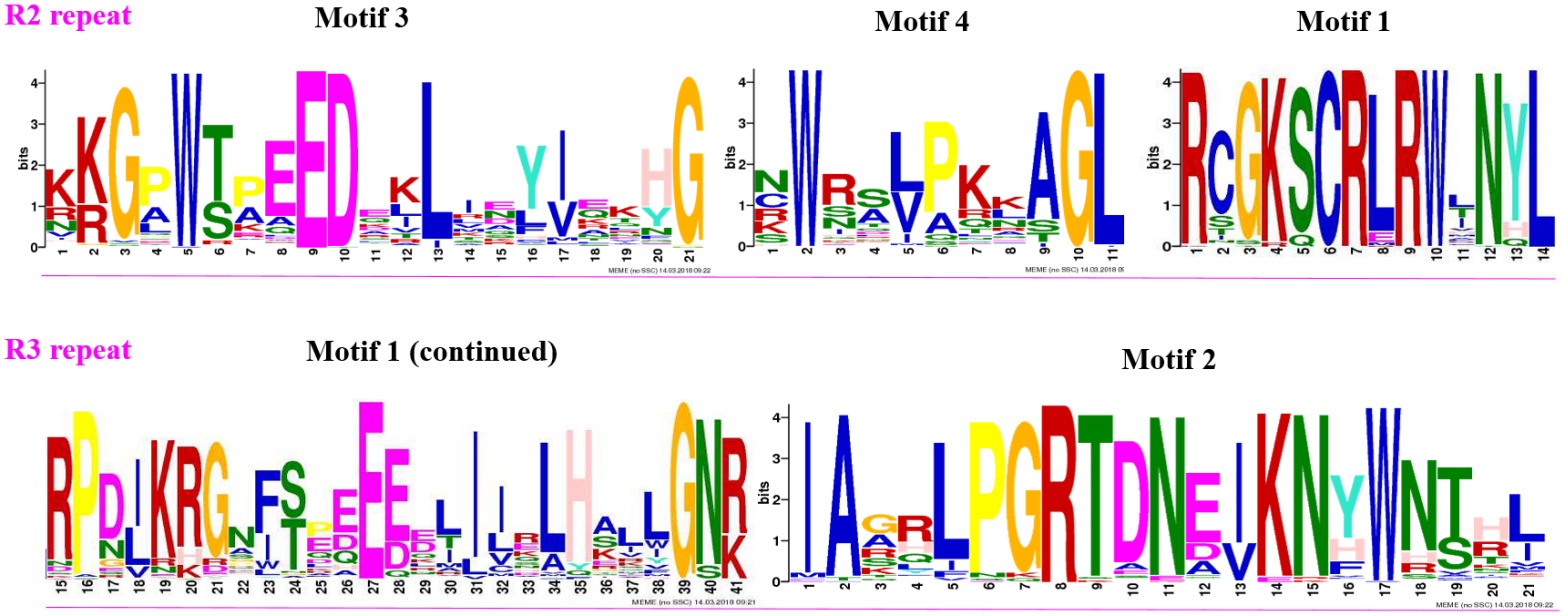
Logo sequences of R2 and R3 repeats of R2R3-MYB subfamily members in peach. The logo sequences of motifs 3, 4, 1, and 2, which together constitute the R2 and R3 repeats in peach. The overall height of each stack represents the conservation of the sequence at that position. The capital letters indicate greater than 50% conservation of amino acids among the MYB domains. The Arabic numerals under the colored capital letters indicate the position of each residue and the width of the motif. Each color of the English letters represents a different type of amino acid residue.

To clearly see the order of MYB proteins and group markers in each motif figure, we arranged them to correspond to their order in the circular phylogenetic tree. A maximum of fifteen motifs that were shared by 132 peach protein sequences (128 R2R3-MYB and 4 R1R2R3-MYB proteins) were first searched in this study, and the corresponding fifteen logo pictures were downloaded from the MEME website (S4 Table). As a result, 83 of the 128 R2R3-MYB protein sequences had four highly conserved motifs and conserved motif orders. These 83 R2R3-MYB protein sequences all had motif 3 (green), motif 4 (blue), motif 1 (red), and motif 2 (sky blue) in that order, containing 21, 11, 41, and 21 amino acids, respectively (Fig 3, S5 and S6 Tables). As the logo composition shows (Fig 4), motifs 3 and 4 and the front part of motif 1 were composed of the R2 repeat, and the back part of motif 1 and all of motif 2 were composed of the R3 repeat. Slightly different from these 83 R2R3-MYB proteins, 9 of 128 R2R3-MYB protein sequences highly conserved motifs 3, 13, 1, and 2 in that order for most members of groups C29 to C31 (Fig 3 and S5 Table). As the logo composition shows (S6 Table), motif 3, motif 13, and the front part of motif 1 were composed of the R2 repeat, and the back part of motif 1 and all of motif 2 were composed of the R3 repeat. There were also several other types of motif connections in this study that were composed of characteristic R2 and R3 repeats (Fig 3 and S6 Table). The residue compositions of logo sequences of R2 and R3 repeats agreed with those reported in other plant species such as soybean [44], sweet orange [30], *Salvia miltiorrhiza* [41], and kiwi [9]. Consistent with previous reports, most of the R2R3-MYB proteins included a highly conserved separated triplet of tryptophan (Trp, W) residues in the R2 repeat of peach (Fig 4), which may form a tryptophan cluster in the three-dimensional HTH structure and play key roles in the aspect of MYB-DNA recognition [40]. Additionally, R2 and R3 repeats were characterized by highly conserved groups of glutamic acid (E)-glutamic acid (E)-aspartic acid (D) residues (EED) and glutamic acid (E)-glutamic acid (E)-glutamic acid (E) residues, respectively (Fig 4). These characteristics were also found in repeats of other plant species such as *Populus* [40], tomato [37], and Chinese white pear [29]. Four (ppa023926m, Prupe.3G219200.1, Prupe.2G307600.1, and Prupe.2G185200.1) of the six members of group C33 (ppa023926m, Prupe.3G219200.1, Prupe.2G307600.1, Prupe.2G185200.1, Prupe.6G349400.1, and Prupe.6G255800.1) had only the R2 repeat in their protein sequences. The motif 3 of Prupe.6G349400.1, which is a part of the R2 repeat, was located behind motif 14; Prupe.6G255800.1 had whole R2 and R3 repeats. Although ppa023926m, Prupe.3G219200.1, Prupe.2G307600.1, and Prupe.2G185200.1 did not have the R3 repeat, these proteins were still clustered into group C33 with AT2G37630 (AtMYB91) (Figs 1 and 3) in this study. Because AT2G37630 belonged to group C33 in a previous report [29], we took that as a classification reference in this study. Similarly, although Prupe.5G230100.1, Prupe.5G147600.1, Prupe.5G047100.1, Prupe.6G210300.1, and Prupe.8G189400.1 only had a part of the R2 and R3 repeats, they were considered MYB proteins and clustered into group C3 because they inhabited the same branch as AT4G17780, which belonged to C3 in the reference [29].

All of the R2R3-MYB members in peach had highly conserved MYB domains at the N-termini of their protein sequences except for those in the last subgroup of C3. In addition to the highly conserved motifs composed of MYB domains (R2 and R3 repeats), other less conserved motifs were also found in some R2R3-MYB proteins in peach (Fig 3 and S6 Table). This finding is consistent with findings in *Salvia miltiorrhiza* [41]. Stracke et al. [26] stated that the C-terminal region next to the MYB domain of most R2R3-MYB proteins usually contains functionally important motifs, although these motifs are less conserved than the MYB domain.

**Fig 5:**
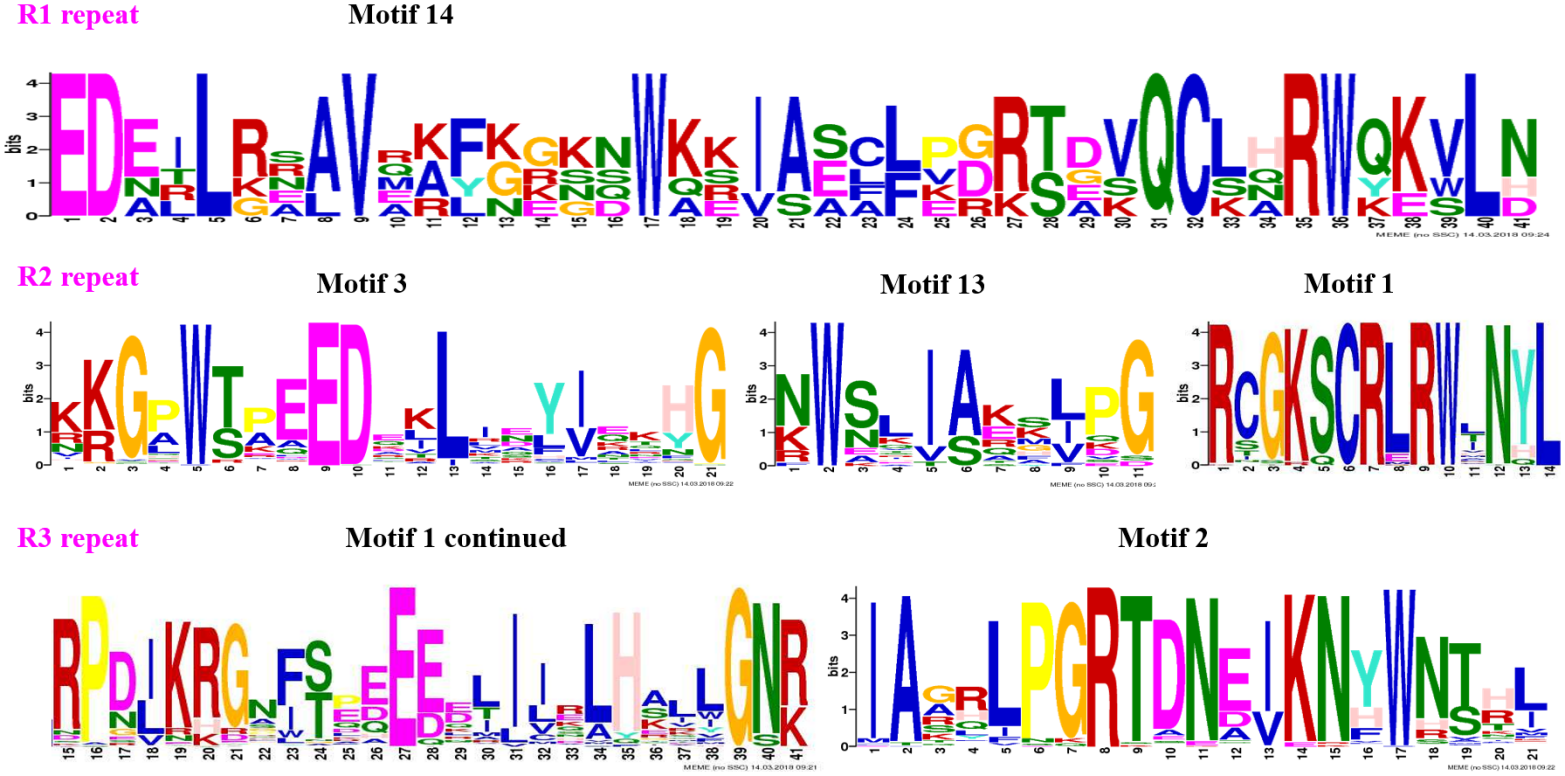
Logo sequences of the R1, R2, and R3 repeats of the R1R2R3-MYB subfamily members in peach. The logo sequences of motifs 14, 3, 13, 1, and 2, which together constitute the R1, R2, and R3 repeats in peach. The overall height of each stack represents the conservation of the sequence at that position. The capital letters indicate greater than 50% conservation of an amino acid among the MYB domains. The Arabic numerals under the colored capital letters indicate the position of each residue and the width of the motif. Each color of the English letters represents a type of amino acid residue.

In terms of the R1R2R3-MYB subfamily in this study (Figs 1 and 3), four peach R1R2R3-MYB members clustered with five *A*. *thaliana* R1R2R3-MYB members in this study (Fig 1). The protein sequences of peach R1R2R3-MYB members contained motifs 14, 3, 13, 1, and 2 in that order except for the lack of motif 14 in Prupe.2G158300.1 (Fig 3). Motif 14 constituted the R1 repeat; R2 was composed of motif 3, motif 13, and a part of motif 1; R3 was composed of the left part of motif 1 and whole motif 2 (S6 Table and Fig 5). The logo sequence of the R1 repeat in this study is a little different from that of the R1 repeat in sweet orange. This difference may be because the differences in peach and orange fruit features lead to differences in aspects of function of R1R2R3-MYB members from peach and orange.

**Fig 6:**
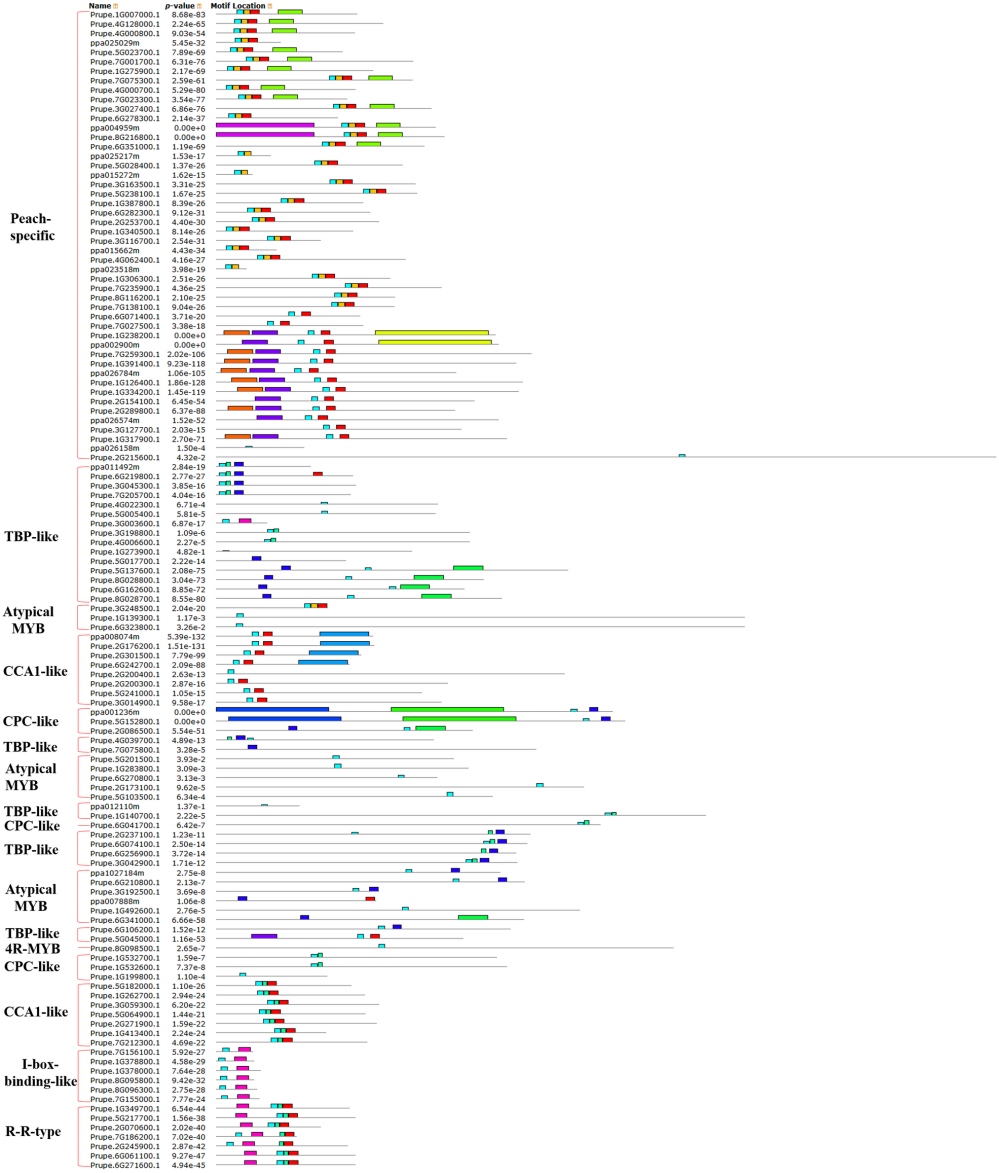
Motif distribution of peach MYB-related (1R-MYB), 4R-MYB, and Atypical-MYB subfamily proteins. Motifs of the 1R-MYB, 4R-MYB, and Atypical-MYB subfamily proteins were analyzed using the MEME web server. Fifteen colors of blocks represent the 15 types of motifs. The length of the gray line indicates the length of a sequence relative to those of all the other sequences. The position of each block indicates the location of a motif with a matching sequence. The red brace on the right marks the members of each group of the 1R-MYB, 4R-MYB, and Atypical-MYB subfamilies in peach.

Although many comprehensive motif analyses of the R2R3-MYB proteins have been conducted, a similar comprehensive analysis of the MYB-related (1R-MYB) subfamily is limited. A maximum of fifteen motifs shared by the remaining 124 of 256 MYB protein sequences were then searched for in this study, and the corresponding fifteen logo pictures were downloaded from the MEME website (S7 Table). Members of the MYB-related (1R-MYB) subfamily in peach were divided into five common groups that also exist in other plant species and a novel group (Peach-specific). All 48 members of the Peach-specific group contained conserved motifs 2 and 1 in that order, which jointly composed the R1 repeat (S8 Table and Fig 6). The CCA1-like group with 8 and 7 members distributed in the two parts in the motif figure (Fig 6). All eight members of the first part of the CCA1-like group except Prupe.2G200400.1 had conserved motifs 2 and 1 in that order, and this arrangement composed an imperfect R1 repeat. All of the 7 members of the second part of the CCA1-like group had conserved motifs 2, 12, and 1 in that order, which composed a perfect R1 repeat (Fig 6 and S8 Table). These results suggest that the members of the CCA1-like group are characterized by having at least motif 2 (sky blue) and motif 1 (red). Six members of the I-box-binding-like group had motifs 2 and 8 in that order, while 7 members of the R-R-type group had motifs 8, 2, 12, and 1 in that order. The motifs 2 and 8 of all 6 members of the I-box-binding-like group composed an imperfect R1 repeat in peach in this study, which was different from the motifs in the logo sequence of the I-box-binding-like group in *A*. *thaliana* [27]. The motifs 2, 12, and 1 of all 7 members of the R-R-type group composed an R1 repeat of peach in this study, which is also different from a report of *A*. *thaliana* sequences. In *A*. *thaliana*, members of the R-R-type group were divided into R-R (A) and R-R (B) subgroups. Additionally, the logo sequence of members within the R-R-type group was not particularly similar to that of the R1 repeat in *A*. *thaliana* [27]. Members of the first two groups of Atypical-MYB subfamily proteins seem to have only one motif, motif 2, which is the front part of the R1 repeat (Fig 6). Members of the third Atypical-MYB group have an imperfect R1 repeat (Fig 6).

Only one member (Prupe.8G098500.1) of the 4R-MYB subfamily was identified in peach (Fig 6), and it had one motif, motif 2. However, when we submitted the protein sequences of Prupe.8G098500.1 and its unique homologous 4R-MYB protein (AT3G18100.1) in *A*. *thaliana* to the MEME website, the results showed that both sequences had four highly conserved motifs (S9 Table). The protein sequence of AT3G18100.1 had motifs 5, 3, 1, and 4 in that order, while the protein sequence of Prupe.8G098500.1 had motifs 3, 5, 1, and 4 in that order. The logo sequences of these four motifs are less conserved than those of the R1, R2, and R3 repeats in this study. This finding is consistent with the finding in *A*. *thaliana* that members of the 4R-MYB subfamily contained four R1/R2-like repeats [7].

### The exon/intron structures of *MYB* genes

**Fig 7:**
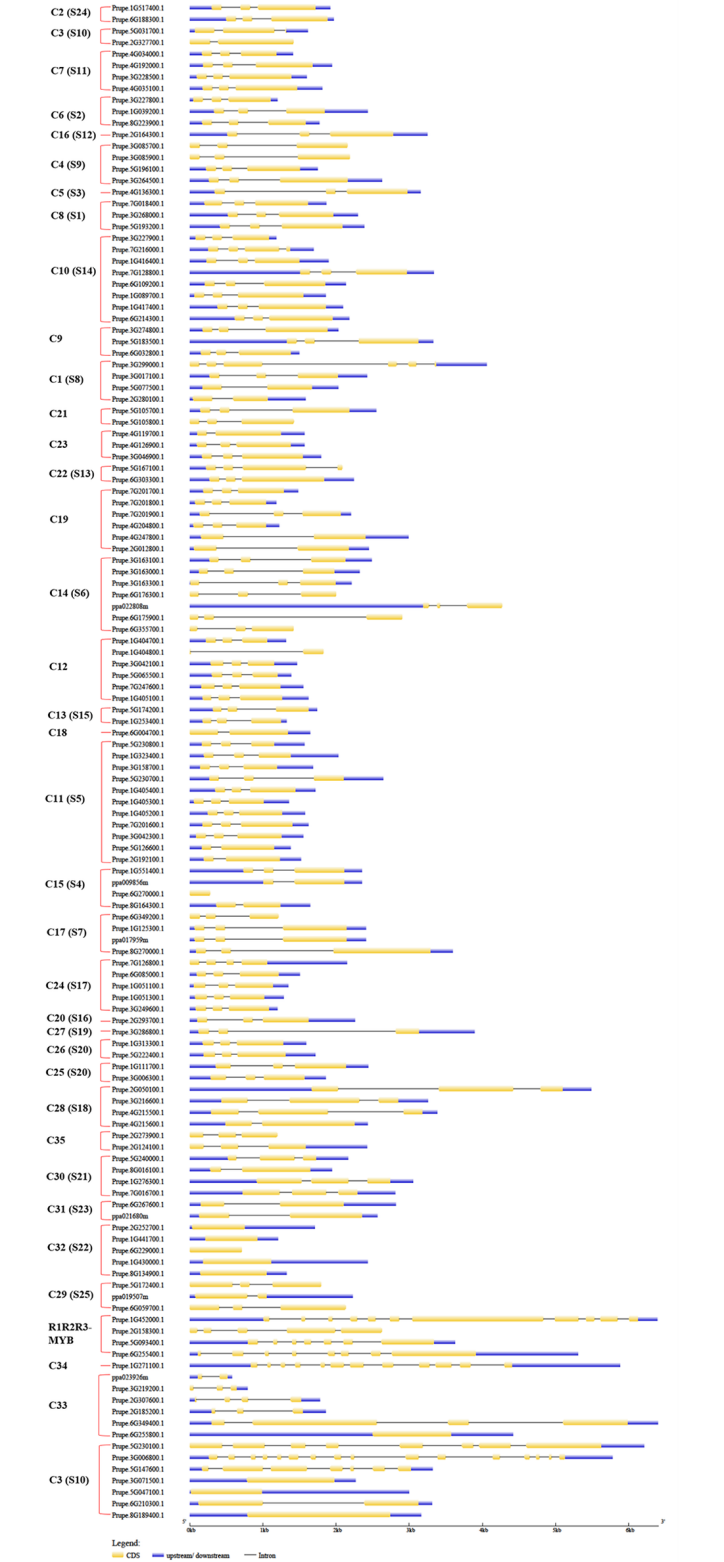
The locations and lengths of exons and introns of peach *R2R3-MYB* and *R1R2R3-MYB* subfamily genes. The exons and introns are presented as filled yellow sticks and thin gray single lines, respectively. Upstream and downstream regions are represented by dark blue bars at the two ends of sequences. The red brace on the right marks the members of each group of the R2R3-MYB and R1R2R3-MYB (3R-MYB) subfamilies in peach.

**Fig 8:**
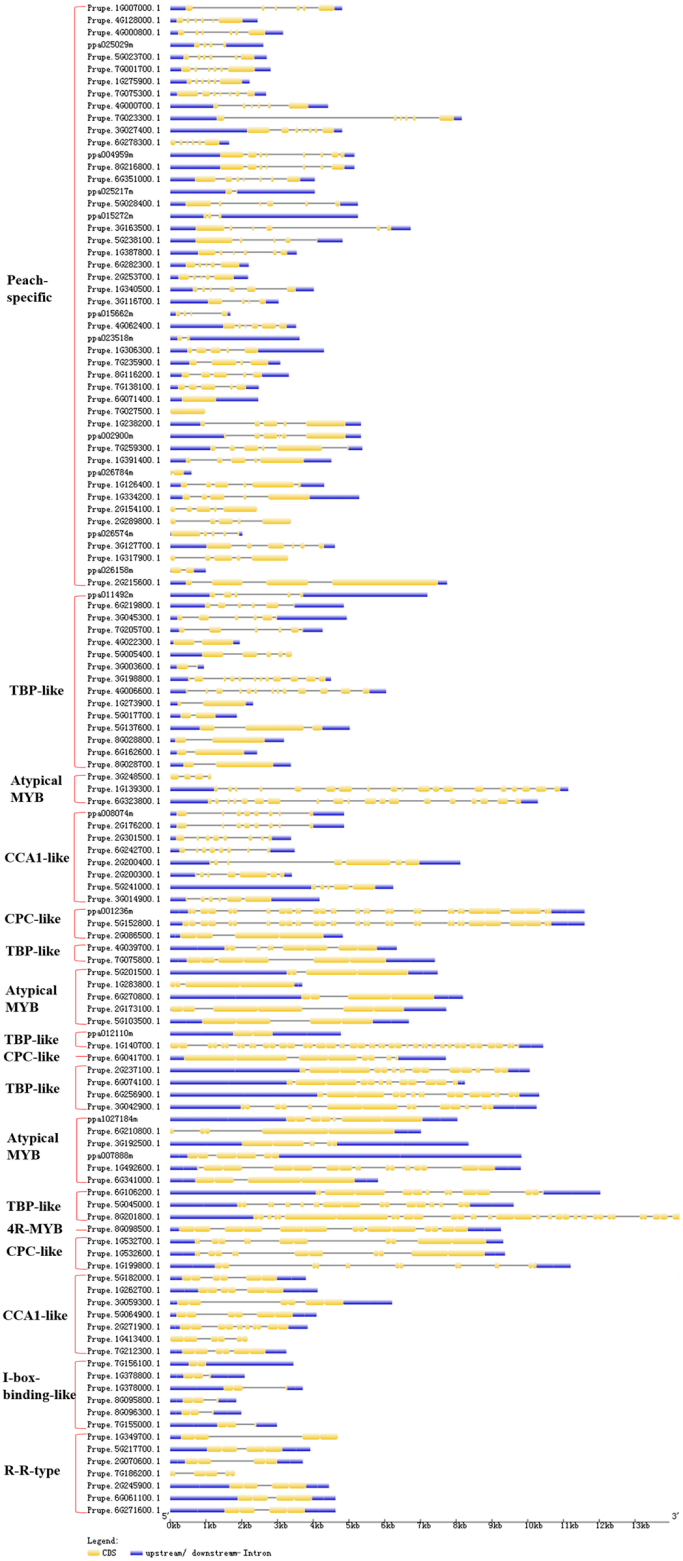
The locations and lengths of exons/introns of peach 1R-MYB, 4R-MYB, and Atypical-MYB subfamily genes. The exons and introns are presented as filled yellow sticks and thin gray single lines, respectively. Upstream and downstream regions are represented by dark blue bars at the two ends of sequences. The red brace on the right marks the members of each group of the 1R-MYB, 4R-MYB, and Atypical-MYB subfamilies in peach.

Similar to the patterns in motif distribution, 256 peach *MYB* genes were separated into two exon/intron schematic structures using the GSDS tool (Figs 7 and 8). In this study, the order of genes in each exon/intron schematic structure is the same as in the circular phylogenetic tree (Figs 1 and 2). The majority of *R2R3-MYB* genes in groups C1 to C30 had three exons and two introns, with the blue regions upstream and downstream of the two ends of the sequences (Fig 7). Surprisingly, these three exons all included two short exons and one relative long exon, and the first short exon and the long exon tightly connected with upstream and downstream regions, respectively (Fig 7). The *R2R3-MYB* genes of group C31 had two exons and one intron, with the blue regions upstream and downstream of the two ends of the sequences, while *R2R3-MYB* genes of group C32 had only one exon and no introns but had blue regions upstream and downstream of the two ends of the sequences with one exception (Prupe.6G229000.1). The exon/intron structure of *MYB* genes in groups C33 and C34 and the last clade of C3 were not conserved among the members of each group in this study. The exon numbers of four members of subfamily R1R2R3-MYB ranged from 5 to 11 and were more than those of members of the R2R3-MYB subfamily other than Prupe.3G006800.1 and Prupe.5G230100.1, which were in the last clade of group C3.

Similar to the conservation of motif composition among the members of each group of subfamily R2R3-MYB, the schematic structures of exons/introns of the members of 1R-MYB, Atypical-MYB, and 4R-MYB subfamilies highly conserved in the numbers and lengths of exons/introns among the members of each group. The exon/intron structures of *MYB* genes in the same subfamily were highly conserved in *B*. *distachyon* [10] and kiwi [9]. Two exons connected by one very short intron were found for the members from TBP-like (the second) and R-R-type groups (Fig 8). Combining the motif distributions (Figs 5 and 6) with the schematic exon/intron structures of MYB superfamily members (Figs 7 and 8), illustrated that the motif distribution structure correlated with the exon/intron structure. Consistent with previous findings, MYB TFs in each group presented similar patterns of exons/introns and conserved motifs, suggesting that these conserved features play crucial roles in group-specific functions [8]. Taken together, the results suggest that the conservation of motifs and exon/intron structure are evolutionarily conserved and functionally important; however, their underlying mechanism of conservation among divergent species is currently unknown.

### Distribution on the chromosome and synteny analysis of *MYB* genes

**Fig 9:**
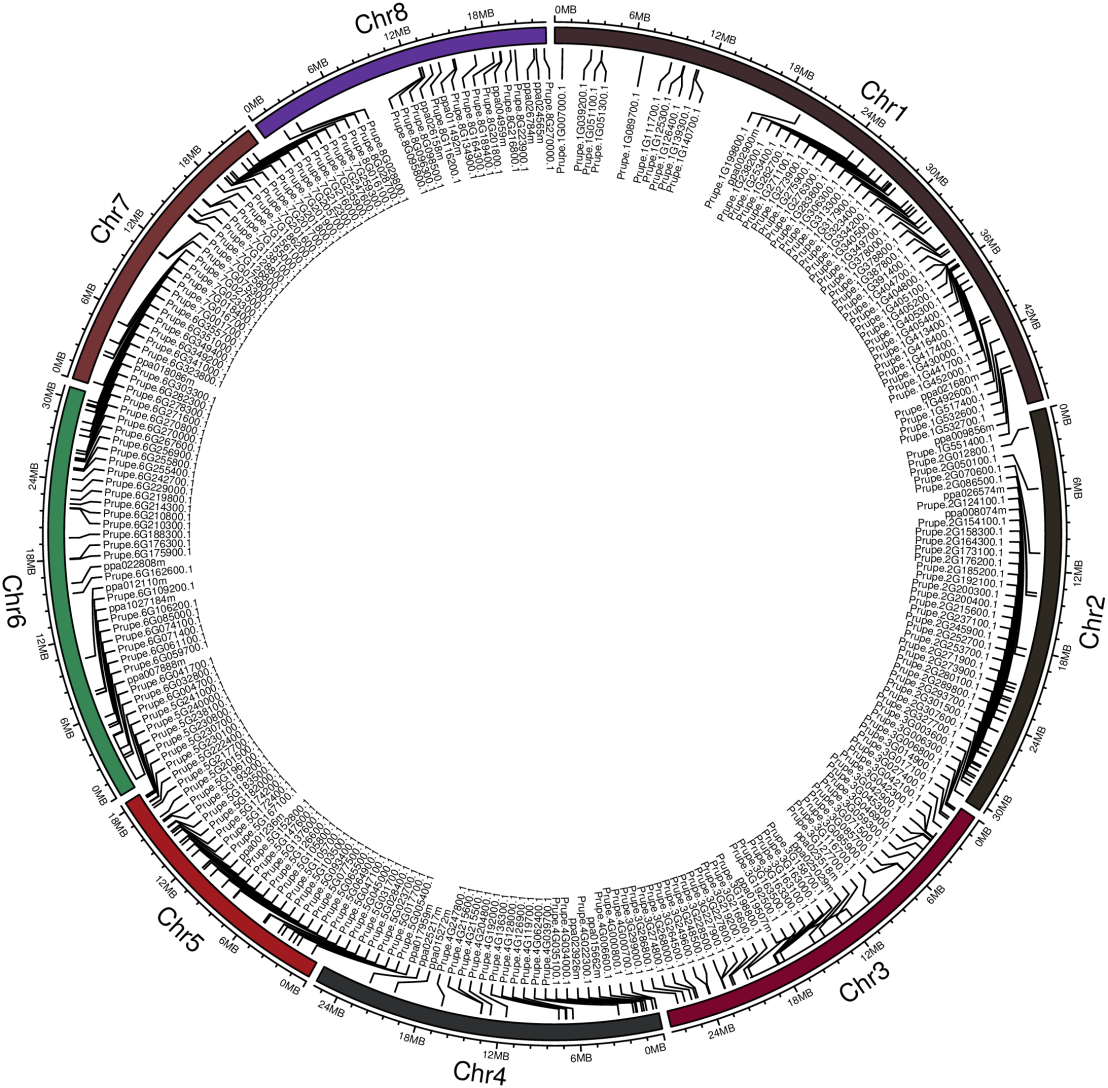
Distribution of *MYB* superfamily genes on the eight chromosomes of peach. The chromosomal location of each MYB gene was mapped to each chromosome using the circlize package of R (v0.4.3) through uploading the start and end information of each gene ID on the corresponding chromosome, as well as the length of each chromosome.

Peach has eight chromosomes, shown ranging from Chr1 to Chr8 (Fig 9). Combined the peach *MYB* gene location on each chromosome (S2 Table) with length of each chromosome obtained from the GDR, the chromosomal location of each *MYB* gene was mapped to each chromosome using the circlize package of R to intuitively display their orders and positions. The 256 *MYB* genes (50, 29, 39, 22, 35, 40, 23, and 18) unevenly distributed on chromosomes 1 to 8 of the peach genome (S2 Table and Fig 9). Chromosome 1 is the longest among the eight chromosomes in peach and had the most *MYB* genes. The *MYB* genes on chromosome 3 were relatively well distributed. The *MYB* genes on chromosomes 6 to 8 were distributed more densely on the two ends of each chromosome than on the middle part of the chromosome. A similar pattern was also reported in tomato [38], sweet orange [30], *B*. *napus* [8]. Most of the chromosomes within a given plant species have *MYB* genes on the two ends of each chromosome, and a low percentage of chromosomes within a given plant species have the *MYB* genes on the middle part of the chromosome.

**Fig 10:**
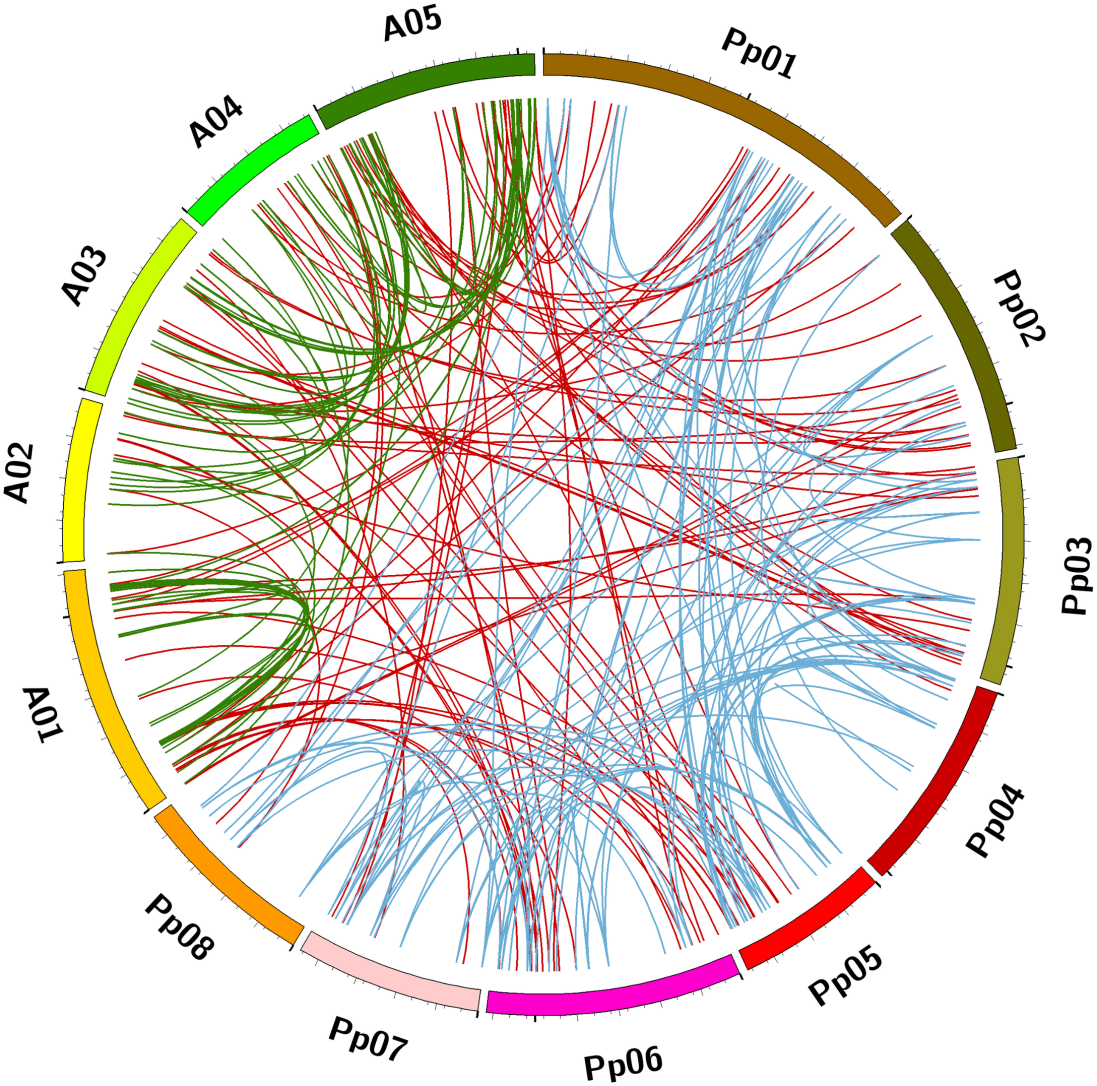
The syntenic relationships of the *MYB* superfamily genes in peach and *A*. *thaliana*. Eight chromosomes of peach (Pp01–Pp08) and five chromosomes of *A*. *thaliana* (A01–A05) were mapped in different colors. The red lines connected the *MYB* genes in peach and *A*. *thaliana* that were orthologous. The blue and green lines connected the paralogous *MYB* genes in peach and *A*. *thaliana*, respectively.

To further intuitively display and illustrate the diversification and evolutionary conservation among MYB superfamily members, syntenic mapping was performed to identify *MYB* genes between peach and *A*. *thaliana* that are orthologs as well as the paralogous *MYB* genes in peach or *A*. *thaliana* using OrthoMcl and Circos tools. As a result, 81 pairs of *MYB* genes in peach and *A*. *thaliana* that are orthologs were identified among the 256 *MYB* genes in peach and 198 *MYB* genes in *A*. *thaliana* (Fig 10 (red line) and S10 Table). Among the orthologous gene pairs of peach and *A*. *thaliana*, each *A*. *thaliana MYB* gene had only one orthologous gene in peach (S10 Table). Five of sixteen *MYB* genes on chromosome 1 of peach were orthologous to five *MYB* genes on chromosomes 1 and 5 (A01 and A05) of *A*. *thaliana*, respectively, and the remaining six peach *MYB* genes were orthologous to three, two, and one *MYB* genes on chromosomes 4, 3 and 2 of *A*. *thaliana*, respectively. Six of thirteen *MYB* genes on chromosome 2 of peach were orthologous to six *MYB* genes on chromosome 2 of *A*. *thaliana*, and the remaining seven peach *MYB* genes were orthologous to four, one, and two *MYB* genes on chromosomes 3, 2, and 1 of *A*. *thaliana*, respectively. Five, four, two, and four of fifteen *MYB* orthologous genes on chromosome 3 of peach had matching numbers of orthologs on chromosomes 5, 3, 2, and 1 of *A*. *thaliana*. On peach chromosome 4, only one gene (Prupe.4G035100.1) had an orthologous *MYB* gene (AT4G21440) in *A*. *thaliana*. Five, one, three, and two of eleven *MYB* genes on chromosome 5 of peach had their orthologs on chromosomes 1, 3, 4, and 5 of *A*. *thaliana*, respectively. Five, two, four, one, and five of seventeen *MYB* genes on chromosome 6 of peach had their orthologs on chromosomes 1 to 5 of *A*. *thaliana*, respectively. One, one, and three of five *MYB* genes on chromosome 7 of peach had their orthologs on chromosomes 1, 1, and 3 of *A*. *thaliana*, respectively. Two and one of three *MYB* genes on chromosome 8 of peach had their orthologs on chromosomes 2 and 4 of *A*. *thaliana*, respectively.

These results indicated that *MYB* genes on chromosomes 1 to 8 of peach aredispersed similarly to *MYB* genes on the five chromosomes of *A*. *thaliana*. Peach *MYB* genes on chromosomes 1 to 8 had relative low-similarity orthologs with *MYB* genes on chromosome 2 of *A*. *thaliana*. No other special rule was found for orthologous *MYB* genes in *A*. *thaliana* and peach in terms of their distribution on chromosomes.

Additionally, 146 pairs of paralogous *MYB* genes were identified on eight chromosomes of peach and were linked by blue lines (Fig 10 and S11 Table). For 198 *MYB* genes of *A*. *thaliana*, 66 pairs of paralogous *MYB* genes were identified on five chromosomes, as shown in green lines (Fig 10 and S11 Table). Each gene in peach had one to six paralogous *MYB* genes; for example, both *Prupe*.*1G007000*.*1* and *Prupe*.*1G405100*.*1* had six paralogous *MYB* genes. Similarly, each gene in *A*. *thaliana* had one to three paralogous *MYB* genes; for example, both *AT1G56650* and *AT1G18570* had three paralogous *MYB* genes. This point was also reported in previous papers [13,29]. The 146 pairs of paralogous *MYB* genes within peach were thus composed of 115 peach *MYB* genes. In accordance with a previous finding of *B*. *distachyon* [10], mojority of the paralogous *MYB* pairs in peach belong to the same group as their original genes. For example, three *MYB* genes paralogous to *Prupe*.*1G405400*.*1* were divided into the group C11 (Fig 1 and S2 Table). According to these results, we deduced that the orthologous *MYB* pairwise between peach and *A*. *thaliana* probably had conservation during evolution and evolved from a common ancestor. The selective pressure might drive genes to be in close proximity during the process of evolution. Furthermore, there was possible functional relatedness between each orthologous pairwise.

### Physicochemical properties of MYB proteins

The molecular weights of 256 peach MYB proteins ranged from 7776.11 to 222179.63 Da, with an average of 44407.54 Da (S12 Table). The calculated pI values ranged from 4.28 to 11.23, with an average of 7.15. In total, 113 of 256 MYB proteins had pI values higher than the average (7.15). The grand average of hydropathy (GRAVY) values of 256 MYB proteins ranged from -1.24 (moderately soluble) to 0.018 (highly soluble). The mean GRAVY value was -0.72, which is similar to that of the MYB proteins in *B*. *napus* [8]. The instability index of 256 peach MYB proteins ranged from 30.27 (Prupe.5G023700.1) to 91.89 (Prupe.2G185200.1), and the average value was 53.99, which is also very similar to that (54.0) of MYB proteins in *B*. *napus* [8].

### Analysis of peach *MYB* gene expression

**Fig 11:**
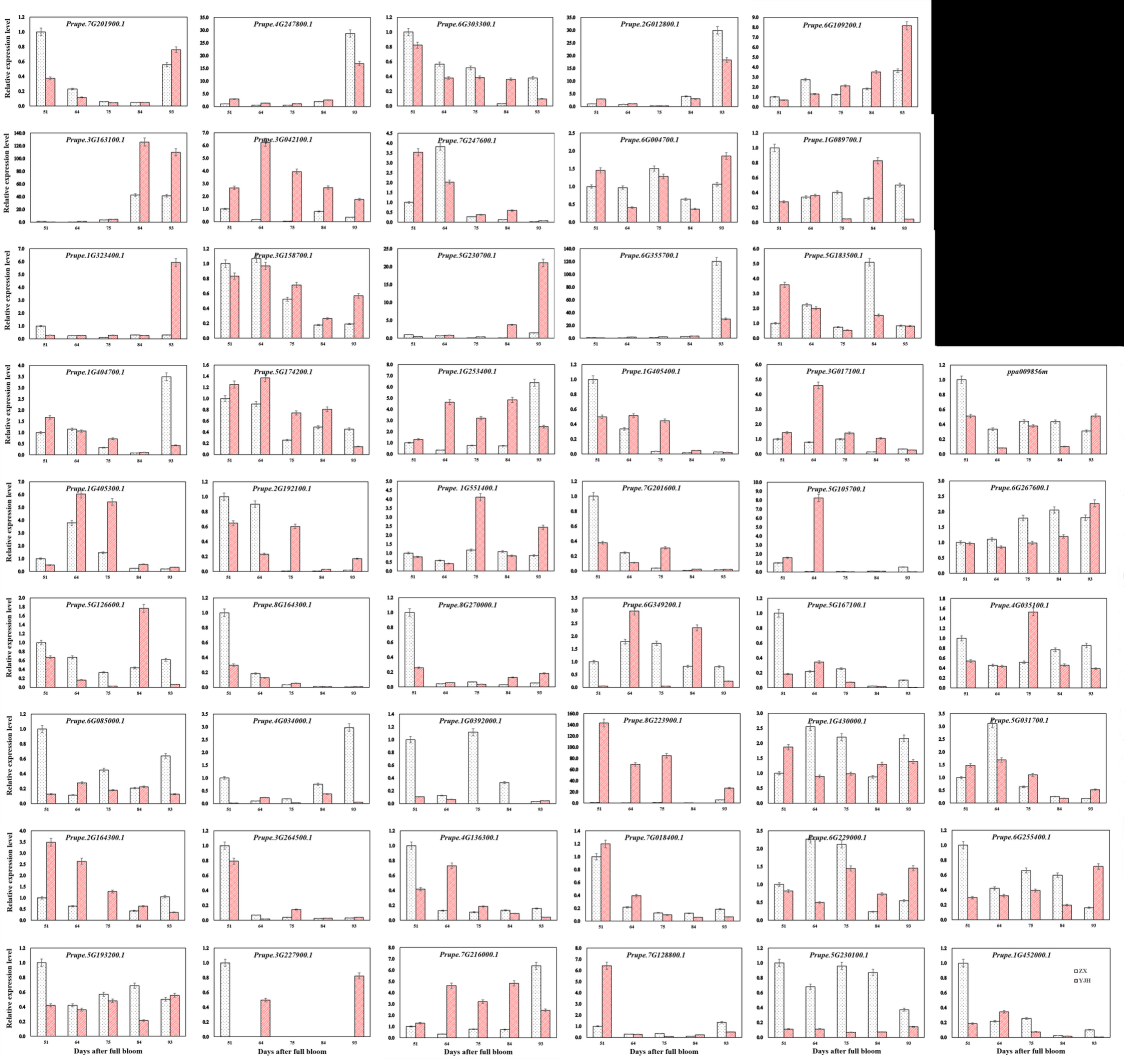
Relative expression levels of selected 51 *MYB* genes in peach fruits of the two cultivars. Error bars represent standard errors from three independent replicates.

*R2R3-MYB* subfamily genes are involved in anthocyanin biosynthesis and the pigmentation of plants [45]. The expression patterns of genes can provide important clues for the prediction of gene function. To assess the potential regulatory roles of *R2R3-MYB* and *R1R2R3-MYB* subfamily genes in generating the red flesh of peach fruits, we analyzed the expression levels of 49 *R2R3-MYB* and two *R1R2R3-MYB* genes in the white-fleshed fruit of ZX and the red-fleshed fruit of YJH sampled 51, 64, 75, 84, and 93 DAFB (Fig 11). The relative expression levels of *Prupe*.*2G164300*.*1*, *Prupe*.*7G018400*.*1*, *Prupe*.*6G303300*.*1*, and *Prupe*.*8G164300*.*1* in the fruit of YJH decreased gradually as the fruit developed from young fruit until they were harvested. The expression of *Prupe*.*2G164300*.*1* in the fruit of ZX was lower than that in the fruit of YJH from 51 to 84 DAFB. The expression level of *Prupe*.*6G303300*.*1* in the fruit of ZX was higher than that in the fruit of YJH from 51 DAFB to harvest with an exception of fruit at 84 DAFB.

Among these 51 *MYB* genes, *Prupe*.*7G201900*.*1*, *Prupe*.*4G247800*.*1*, and *Prupe*.*2G012800*.*1*, belonging to group C19 of the R2R3-MYB subfamily, had similar expression patterns at most developmental stages. *Prupe*.*4G247800*.*1* and *Prupe*.*2G012800*.*1* had almost the same expression patterns. Another example in this study is *Prupe*.*1G323400*.*1* and *Prupe*.*5G230700*.*1* of group C11, which also had similar expression patterns, while *Prupe*.*1G405400*.*1* and *Prupe*.*7G201600*.*1* had similar expression patterns that were different from those of *Prupe*.*1G323400*.*1* and *Prupe*.*5G230700*.*1*. *Prupe*.*3G042100*.*1*, *Prupe*.*7G247600*.*1*, and *Prupe*.*1G404700*.*1* of group C12 also shared similar expression patterns. Similar expression patterns were also found for *Prupe*.*3G163100*.*1* and *Prupe*.*6G355700*.*1* of group C14. These results illustrated that some members of the same group (termed a subfamily in some papers) of the peach R2R3-MYB subfamily showed similar expression patterns. This result is in accordance with the previous findings in orange [30]. These results can be explained by the fact that most members of a given group (or subgroup or subfamily) had more similarities in the MYB domain and gene structure as shown in peach.

*Prupe*.*3G163100*.*1*, *Prupe*.*1G323400*.*1*, *Prupe*.*4G247800*.*1*, *Prupe*.*5G230700*.*1*, *Prupe*.*2G012800*.*1*, and *Prupe*.*6G355700*.*1* had the highest expression levels in the mature fruits of YJH, and these levels were relatively stable from 51 to 84 DAFB with an exception of *Prupe*.*3G163100*.*1* on 84 DAFB. Additionally, the expression levels of *Prupe*.*3G163100*.*1*, *Prupe*.*1G323400*.*1*, *Prupe*.*4G247800*.*1*, and *Prupe*.*5G230700*.*1* were lower in mature fruits of ZX than in mature fruits of YJH. In contrast, the expression level of *Prupe*.*6G355700*.*1* was higher in mature fruits of ZX than in mature fruits of YJH. Overall, the expression level of *Prupe*.*6G109200*.*1* was higher in green ZX fruits than in green YJH fruits from 51 to 64 DAFB, while from 75 to 93 DAFB, the expression level of *Prupe*.*6G109200*.*1* was higher in YJH than in ZX. Overall, the expression level of *Prupe*.*6G267600*.*1* in ZX increased gradually as the fruit developed from 51 to 84 DAFB but slightly decreased at harvest. *Prupe*.*6G267600*.*1* expression levels were stable from 51 to 75 DAFB in the YJH fruits and then increased gradually until harvest, at which point they were finally higher than the expression level in mature ZX fruits. The expression level of *Prupe*.*5G230100*.*1* was far higher in the fruit of ZX than in the YJH fruits in young to mature fruits; it was relatively low and stable throughout the development of YJH fruits. *Prupe*.*8G223900*.*1* displayed a high expression level in the YJH fruits and was almost undetectable in the ZX fruits from 51 to 84 DAFB. The high expression level divergence of other *MYB* genes at different developmental stages in two cultivars reflected the complexity of the regulatory functions of *MYB* genes in their fruits. Some *R2R3-MYB* genes in this study were differentially expressed at five different stages of fruit development, and no special rule was found to describe expression during the fruit development of each cultivar. This finding suggested that *R2R3-MYB* genes might play an important role in the fruit development and/or ripening of peach through involvement in various aspects of physiological and physiochemical processes, combined with interactions with other genes.

Some *R2R3-MYB* genes in peach fruit have been studied functionally. For example, the peach *MYB7* of subfamily R2R3-MYB was reported to regulate the synthesis of proanthocyanidins [46], which form a group of natural phenolic compounds that greatly affect both the flavor and nutritious value of fruit. Similarly, *MYB10* was isolated from peach, and patches of anthocyanin were most apparent with *MYB10* when *AtbHLH2* was included as a partner [13]. The expression profiles of six peach *R2R3-MYB* genes, including *MYB10*.*1* (ppa026640m), *MYB10*.*2* (ppa016711m), *MYB10*.*3* (ppa020385m), *MYB12* (ppa004560m), *MYB44* (ppa008450m), and *MYB108* (ppa015973m), were highly correlated with those of the anthocyanin biosynthesis genes in fruits [47]. Similarly, previous reports in other fruit tree species also demonstrated that MYB TFs such as the MYBs in Chinese bayberry [48], apple [49], litchi [50] and grape [51] are critical for anthocyanin biosynthesis. In addition to functioning in anthocyanin biosynthesis in the peach fruit, *R2R3-MYB* genes werereported to function in other aspects of fruit development. The transition from S3 to S4 was paralleled by changes in the expression of *MYB* genes assessed by microarray analysis [52]. Vendramin provided strong evidence that the peach *MYB25* gene (*ppa023143m*) acts as a positive regulator of trichome formation in peach fruit [53].

In addition to their roles in fruit, *R2R3-MYB* genes were also reported to be involved in the development of flowers and leaves. For example, *MYB10* might be involved in controlling the biosynthesis of anthocyanins in red peach leaves [54]. The anthocyanin accumulation in peach flowers was coordinately regulated by a set of *R2R3-MYB* genes [55]. The function of the remaining *MYB* genes in peach should be verified further through experiments.

## Conclusions

The present study was the first genome-wide detailed analysis of the *MYB* superfamily genes in peach. In total, this study yielded a set of 256 *MYB* superfamily genes containing 128 *R2R3-MYB* subfamily genes (2R-MYB), 4 *R1R2R3-MYB* subfamily genes (3R-MYB), 109 *MYB-related subfamily* genes (1R-MYB), one *4R-MYB* subfamily gene, and 14 *Atypical-MYB* subfamily genes. The 128 *R2R3-MYB* subfamily genes in peach were clustered into 35 groups, and the 109 *MYB-related* subfamily genes were further clustered into six groups: the CCA1-like, CPC-like, TBP-like, I-box-binding-like, R-R-type, and Peach-specific groups. The members of each group within the R2R3-MYB or MYB-related subfamily in peach were highly conserved in terms of their motif composition and exon/intron organization. The logo sequences of the R2 and R3 repeats of most R2R3-MYB subfamily members were highly conserved with those in several other plant species. The phylogenetic relationship between MYBs in peach and *A*. *thaliana* indicated that peach and *A*. *thaliana* displayed both the conserved and specialized evolution of MYB members. Additionally, the 256 *MYB* genes unevenly distributed on chromosomes 1 to 8 of the peach genome. Eighty-one pairs of *MYB* genes in peach and *A*. *thaliana* wereidentified as orthologs among 256 *MYB* genes in peach and 198 *MYB* genes in *A*. *thaliana* in this study. In addition, 146 pairs of paralogous *MYB* genes were identified among the eight chromosomes of peach. The expression levels of some genes of 51 *MYB* genes selected for qRT-PCR analysis tended to decrease or increase in red-fleshed fruit development, while the expression patterns of some genes followed no clear rules during the five developmental stages of the fruits. Further studies are needed to unravel *MYB* function in peach, which may lead to better insights into the molecular mechanisms that regulate the growth and development of peach fruit.

## Supporting information

S1 Table Sequences of primers designed for qRT-PCR analysis of 51 selected *MYB* superfamily genes in peach. (XLSX)

S2 Table Summary of *MYB* superfamily genes in peach. Yellow and slight blue represent the members of the first phylogenetic tree; the orange and green represent the members in the second phylogenetic tree.
(XLSX)

S3 Table List of 131 MYB proteins/genes including 126 R1R2R3-MYBs and 5 R2R3-MYBs in *Arabidopsis*. (XLSX)

S4 Table Logo sequences of each motif of 15 conserved motifs shared by proteins of subfamilies R1R2R3-MYB and R2R3-MYB in Fig 3. (XLSX)

S5 Table List of 83 and 9 R2R3-MYB proteins with respective conserved motif in peach. (XLSX)

S6 Table Logo sequences of motifs shared by each protein of subfamilies R1R2R3-MYB and R2R3-MYB in Fig 3. (XLSX)

S7 Table Logo sequences of each motif of 15 conserved motifs shared by proteins of subfamilies 1R-MYB, Atypical-MYB, and 4R-MYB in Fig 6. (XLSX)

S8 Table Logo sequences of motifs shared by each protein of subfamilies 1R-MYB, Atypical-MYB, and 4R-MYB in Fig 6. (XLSX)

S9 Table Logo sequences of motifs shared by 4R-MYB proteins of *Arabidopsis* and peach. (XLSX)

S10 Table Summary of *MYB* genes in *Arabidopsis* and peach that are orthologs. Each orthologous pair of peach/*Arabidopsis MYB* genes is listed on a line in Excel and is linked by a red line in Fig 6.
(XLSX)

S11 Table Summary of paralogous *MYB* genes in *Arabidopsis* or peach. Each pair of paralogous *MYB* genes in *Arabidopsis* or peach is listed on a line in Excel and is linked by green or blue lines in Fig 6, respectively.
(XLSX)

S12 Table Summary of physicochemical properties of MYB proteins in peach. (XLSX)

## Abbreviations

CDSs: coding sequences
DAFB: days after full bloom
GDR: Genome Database for Rosaceae
GSDS: Gene Structure Display Server
HMM: hidden markov model
HTH: helix-turn-helix
MEME: Multiple Em for Motif Elicitation
ORF: open reading frame
qRT-PCR: quantitative real-time polymerase chain reaction
*RP II*: *RNA polymerase II*
TAIR: The Arabidopsis Information Resource
TFs: transcription factors
v: version
YJH: Yejihong
ZX: Zhaoxia
